# Postmortem age estimation via DNA methylation analysis in buccal swabs from corpses in different stages of decomposition—a “proof of principle” study

**DOI:** 10.1007/s00414-020-02360-7

**Published:** 2020-07-07

**Authors:** Barbara Elisabeth Koop, Felix Mayer, Tanju Gündüz, Jacqueline Blum, Julia Becker, Judith Schaffrath, Wolfgang Wagner, Yang Han, Petra Boehme, Stefanie Ritz-Timme

**Affiliations:** 1 Institute of Legal Medicine, University Hospital Düsseldorf, 40225 Düsseldorf, Germany; 2 Helmholtz-Institute for Biomedical Engineering, Stem Cell Biology and Cellular Engineering, RWTH Aachen Faculty of Medicine, Aachen, Germany

**Keywords:** Age estimation, DNA methylation, Corpses, Decomposition, Buccal swabs, Postmortem

## Abstract

Age estimation based on the analysis of DNA methylation patterns has become a focus of forensic research within the past few years. However, there is little data available regarding postmortem DNA methylation analysis yet, and literature mainly encompasses analysis of blood from corpses without any signs of decomposition. It is not entirely clear yet which other types of specimen are suitable for postmortem epigenetic age estimation, and if advanced decomposition may affect methylation patterns of CpG sites. In living persons, buccal swabs are an easily accessible source of DNA for epigenetic age estimation. In this work, the applicability of this approach (buccal swabs as source of DNA) under different postmortem conditions was tested. Methylation levels of *PDE4C* were investigated in buccal swab samples collected from 73 corpses (0–90 years old; mean: 51.2) in different stages of decomposition. Moreover, buccal swab samples from 142 living individuals (0–89 years old; mean 41.2) were analysed. As expected, methylation levels exhibited a high correlation with age in living individuals (training set: *r*^2^ = 0.87, validation set: *r*^2^ = 0.85). This was also the case in postmortem samples (*r*^2^ = 0.90), independent of the state of decomposition. Only in advanced putrified cases with extremely low DNA amounts, epigenetic age estimation was not possible. In conclusion, buccal swabs are a suitable and easy to collect source for DNA methylation analysis as long as sufficient amounts of DNA are present.

## Introduction

Epigenetic age estimation using “molecular clocks” [1–4] opens up new opportunities in forensic case work. The basis of such an epigenetic clock is an age-dependent methylation pattern at specific CpG sites. Several models for epigenetic age estimation have been described, which differ regarding number and combination of CpG sites (for review, see [5]). This new approach for age estimation can be used especially in the context of the analysis of crime scene samples [6] as well as for low- or non-invasive age estimation of living individuals based on easily accessible test material such as blood, saliva, or buccal swab samples (e.g., [7–11]).

Another forensic application for epigenetic age estimation may be age at death estimation of unknown deceased. Bekaert and colleagues [12] developed a 4-CpG-model (*ASPA*, *PDE4C*, *ELOVL2* and *EDARADD*) using blood samples from 37 living and 169 deceased individuals. Estimated ages of the deceased were in accordance with those from living individuals. Similar results were reported by Hamano et al. [13] and Naue et al. [14] using two (*ELOVL2* and *FHL2*) [13] and 13 CpG sites (*RPA2*, *F5*, *TRIM59*, *KLF14*, *HOXC4*, *NKIRAS2*, *ZYG11 A*, *MEIS1*, *ELOVL2*, *GRM2*, *LDB2*, *SAMD2*, *DDO*) [14], respectively. Additionally, the work of Naue et al. [14] revealed that methylation levels of several CpG sites were also associated with chronological age in several tissues collected postmortem including muscle, brain, bone and buccal swabs. The applicability of age estimation based on DNA methylation to postmortem tissues was also confirmed by further groups like Dias and colleagues for blood [15], Lee et al. for bone samples [16], Pfeifer et al. for blood [17] and Marquez-Ruiz et al. for teeth [18]. However, most authors either analysed only corpses in early stages of decomposition or did not describe the degree of decomposition in their cases.

Dias et al. [19] just recently pointed to possible postmortem changes of DNA methylation patterns. They investigated 62 blood samples, which were collected within 5 days after death and compared the results to those of samples from 59 living individuals. The authors concluded “that postmortem changes can occur in the methylation levels” and “postmortem changes can alter the methylation status among specific loci”.

Clarifying the raised question of postmortem changes in the DNA methylation pattern is a prerequisite for the application of age estimation based on DNA methylation to postmortem samples. To date, there are no systematic analyses regarding the effects of decomposition on epigenetic age estimation.

This study is intended to contribute to a better understanding of postmortem effects on DNA methylation. It focuses on whether epigenetic age estimation based on the analysis of buccal swabs is possible in cases with advanced postmortem decomposition. Buccal swabs from decedents were taken postmortem during different stages of decomposition. The degree of DNA methylation was assessed from 73 corpses and 142 living individuals for the highly age-associated CpG-1 site (upstream of cg17861230) of *PDE4C* in buccal swab samples.

## Material and methods

### Sample collection and assessment of decomposition

Buccal swabs were collected from 73 deceased individuals (age 0–90, mean: 51.2 years; 68.5% male) in different stages of decomposition and a postmortem interval (precisely known in 42 of the 73 cases) of 1–42 days. An additional 142 samples were taken from healthy living individuals (age 0–89 years, mean: 41.6 years; 41.5% male).

In cases of deceased individuals, macroscopically visible external changes of decomposition were described and the cases subsequently classified based on the proposed scorings by Megyesi et al. [20] (Table 1). Decomposition scores of the heads were used to group samples into the following categories with respect to their degree of decomposition: score 1 for no signs of decomposition (*n* = 21), score 2–3 for early signs of decomposition (*n* = 18), score 4–5 for signs of advanced decomposition (*n* = 22) and a score > 6 for severe signs of decomposition (*n* = 12).

**Table 1:** Scoring sheet for assessment of decomposition (Megyesi et al. [20])

Score	Signs of decomposition
1	Fresh, no discoloration
2	Pink-white appearance with skin slippage, some hair loss
3	Gray to green discoloration of skin
4	Drying of nose, ears and lips
5	Purging of decompositional fluids out of eyes, ears, nose, mouth
6	Muscle tissue brown-black
7	Caving in of facial tissue
8	Bones visible (< 50% of scoring area)
9	Mummification, bones visible > 50% of scoring area

### DNA isolation and pyrosequencing

For genomic DNA isolation, NuceloSpin® Tissue Kit by Macherey-Nagel was used according to the manufacturer’s standard protocol for human tissue with overnight lysis. Extracted genomic DNA was stored at − 20 °C until further analysis. DNA quantity and quality were measured using the Investigator Quantiplex Pro Kit (Qiagen) via real-time PCR (Applied Biosystems™ 7500 Real-Time PCR Systems) following manufacturer’s instructions with default settings. For bisulfite conversion, the EZ DNA Methylation-Gold™ Kit (Zymo Research) was used following manufacturer’s instructions. When possible, the recommended amount of 200–500 ng of input DNA was applied. In some samples, the input was lower due to low original DNA amounts (Table 2). Bisulfite-converted DNA was amplified using the primers described by Weidner et al. [4] using the following thermal cycler conditions: 95 °C, 15 min; 45× cycles (95 °C, 30 s; 52 °C, 30 s; 72 °C, 30 s); 72 °C, 5 min; 4 °C, hold. The length of the subsequent product was 155 bp.

**Table 2:** Postmortem samples: DNA concentrations (ng/μl) and degradation indices (*: samples with DNA quantities too low for further application (≤ 3 ng/μl); score 1, no signs of decomposition, *n* = 21; score 2 + 3, early signs of decomposition, *n* = 18; scores 4 + 5, advanced decomposition, *n* = 22; score > 6, severe signs of decomposition, *n* = 12)

Score 1	ng/μl DNA	Degradation index	Score 2 + 3	ng/μl DNA	Degradation index	Score 4 + 5	ng/μl DNA	Degradation index	Score > 6	ng/μl DNA	Degradation index
L05	31.94	1.91	L03	84.62	1.48	L06	79.08	1.43	L01 *	0.00	Not applicable (degradation)
L08 *	0.03	42.57	L09	12.49	3.11	L15	38.53	6.18	L07 *	0.01	7.43
L12	14.59	1.43	L10	60.82	4.68	L16	60.94	2.31	L14 *	1.12	4.93
L21 *	0.01	6.01	L11	154.96	1.89	L19	102.89	1.22	L33 *	0.01	4.86
L22	27.92	5.32	L13	56.24	1.36	L28 *	0.00	4.34	L64 *	0.19	2.82
L23 *	0.02	12.69	L17	63.28	6.90	L30	70.12	2.13	L65 *	0.01	4.4
L25	12.58	1.32	L18	134.57	1.63	L34	72.12	15.34	L71 *	0.00	4.75
L26	110.62	1.82	L24	12.97	8.14	L36	66.14	8.79	L74 *	0.00	13.02
L27	33.44	1.28	L32	233.64	1.77	L39	45.85	6.64	L76 *	0.00	2.7
L29	66.13	1.63	L35 *	2.00	2.30	L42	3.55	4.28	L79 *	0.01	27.28
L31	16.69	2.30	L40	73.56	1.51	L44 *	1.04	10.54	L80	100.76	3.38
L37	9.65	1.86	L57	303.26	1.59	L45	97.53	1.82	L81 *	0.01	2.56
L43	56.78	1.62	L63	144.22	1.94	L46	33.03	3.58			
L47	16.73	4.42	L69 *	0.00	23.26	L49	59.44	1.41			
L50	74.78	2.07	L75	10.22	2.06	L51	80.71	5.24			
L53	33.71	2.88	L77	93.60	3.62	L56	62.74	3.74			
L54	36.53	1.74	L82	20.51	5.04	L58	122.19	2.75			
L60 *	0.47	1.52	L83 *	0.00	5.00	L61	70.72	3.37			
L70	63.45	2.63				L62	51.11	12.14			
L73	90.10	1.65				L67 *	0.17	6.44			
L78	134.01	2.67				L68	41.27	2.16			
						L72	49.28	2.59			
Mean	39.53	4.83		81.16	4.29		54.93	4.93		8.51	7.10

Subsequently, the converted and amplified DNA was immobilized to 1 μl Streptavidin Sepharose™ HP beads (GE Healthcare). For pyrosequencing, the Pyromark Q24 and Pyromark Q24 Advanced Systems (Qiagen) were used. Sequencing primers were described in Weidner et al. [4]. All samples were measured at least in duplicate. Single values did not differ by more than 3% within one sample; this was also true for comparative measurements using both pyrosequencing systems (Q24, Q24 Advanced).

### Data analysis

The relationship between chronological age and the methylation status of the highly age-associated CpG-1 site of *PDE4C* (upstream of cg17861230; see Weidner et al. [4]) was tested by linear regression and the corresponding correlation coefficients were determined. The samples of living individuals were split into two groups. One group was used as training set (*n* = 71, ages 0–89, mean age: 42 years), the other as validation set *n* = 71, ages 0–85, mean age: 42 years). Corpses with sufficient DNA yields (see Table 2) were treated as a second (postmortem) validation set (*n* = 52, ages 0–88, mean age: 49.5 years). The MADs (mean absolute deviations) of age estimates were calculated as mean of deviations of estimated from chronological ages.

### Cytology of buccal swabs

Buccal swabs were taken from corpses in different stages of decomposition (scores 1, 5, and 7) and smears were prepared on specimen slides. The smears were stained with the Pappenheim method: after drying, the slides were immersed in undiluted May-Grünwald stain for 5 min, rinsed with distilled water, immersed in Giemsa stain diluted with distilled water in a ratio of 1:9 for 15 min before being rinsed again with distilled water. After staining, images of the smears were taken with the Nikon C-TEP3 system with a 100-fold magnification.

## Results

### DNA yields were unexpectedly high in postmortem samples—apart from cases with severe decomposition (score > 6)

DNA quantities varied considerably within both groups (living and deceased) and ranged between < 1.0 and 303.3 ng/μl (Table 2). In several postmortem samples, DNA yields were unexpectedly high, albeit, in the group with severe signs of decomposition (score > 6), only one sample (L80) contained sufficient amounts of DNA for further analysis. The number of samples with low DNA amounts increased with increasing decomposition. Thus, the manufacturer’s recommended input of 200–500 ng of template DNA for bisulfite conversion was only possible for 52 of 73 samples of deceased (Table 2). There was no relationship between degradation indices and DNA yields or macroscopically assessed scores of decomposition for samples of scores below 6 (Table 2). Degradation indices over 10 (indicating a possible degradation) were seen in only few cases in all score groups (2 of 21 cases of score 1, 1 of 18 cases of score 2 + 3, 3 of 22 cases of score 4 + 5, and 2 of 11 cases of score > 6). Samples with high degradation indices neither showed pyrograms of low quality nor values that do not correspond to the age of the individuals.

**Fig. 1:**
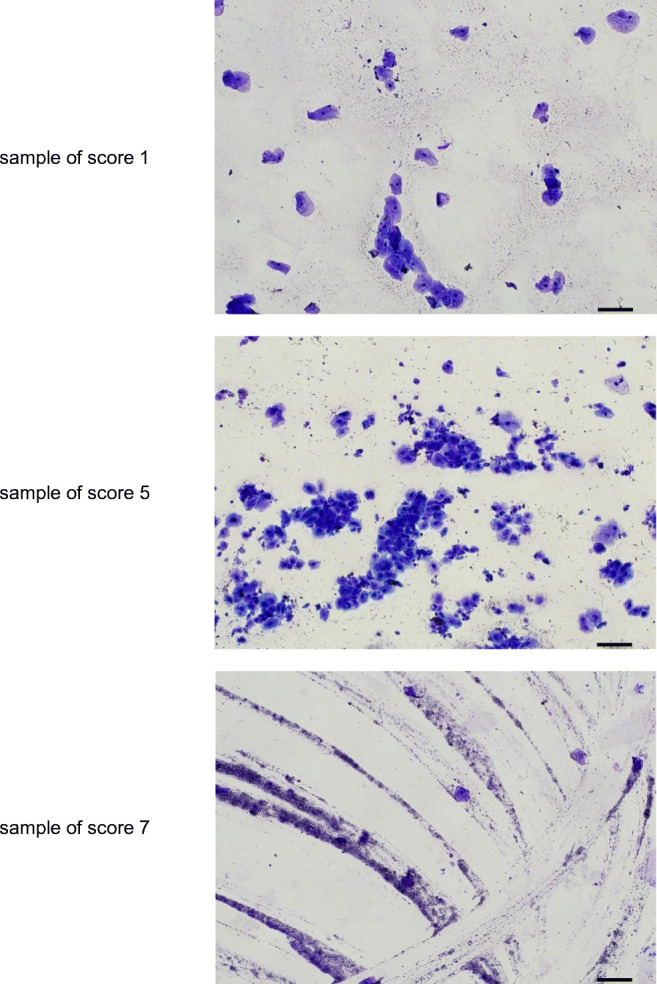
Typical examples for smear cell preparations of buccal swabs, 100× magnification, scale bars represent 100μm

Smear cell preparations from buccal swabs showed a significant increase of buccal cells in preparations from corpses with signs of decomposition up to score 5, as compared to corpses without signs of decomposition (score 1) (Fig. 1). However, in corpses with advanced decomposition, nearly no intact buccal cells could be found (example in Fig. 1: score 7).

### Ante- and postmortem samples exhibit a similar and close correlation between DNA methylation and chronological age

Statistical analyses of the training set (71 living individuals) showed a close relationship between age and DNA methylation (*r*^2^ = 0.87), which can be described by the following equation:

estimated age = (methylation of CpG-1 in PDE4C − 9.5485) / 0.4797

**Fig. 2:**
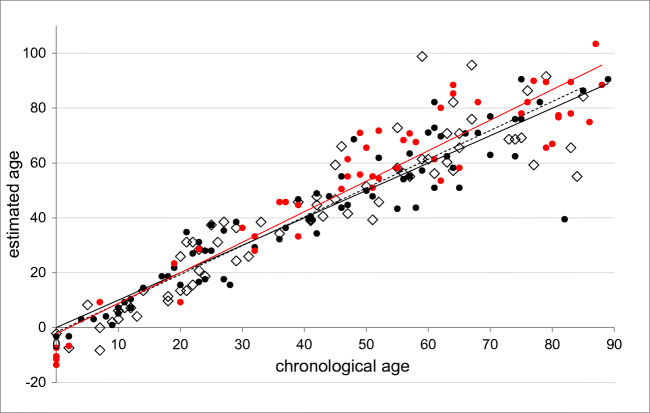
Estimated vs.chronological age of living individuals; black dots and black regression line: training set (*n* = 71, *r*^2^ = 0.87); white rhombi and dashed regression line: validation set living individuals (*n* = 71, *r*^2^ = 0.85, MAD = 7.8 years); red dots and red regression line: validation set corpses (*n* = 52, *r*^2^ = 0.90, MAD = 9.1 years)

Application of this formula to both validation groups (71 living individuals, 52 corpses) revealed a similar and close relationship between chronological and estimated age for all groups (Fig. 2, validation set living: *r*^2^ = 0.85, validation set corpses: *r*^2^ = 0.90), the MADs were 7.8 years for the validation set of living individuals, and 9.1 years for the postmortem samples.

#### The state of decomposition does not affect the scattering of methylation levels in the postmortem samples

**Fig. 3:**
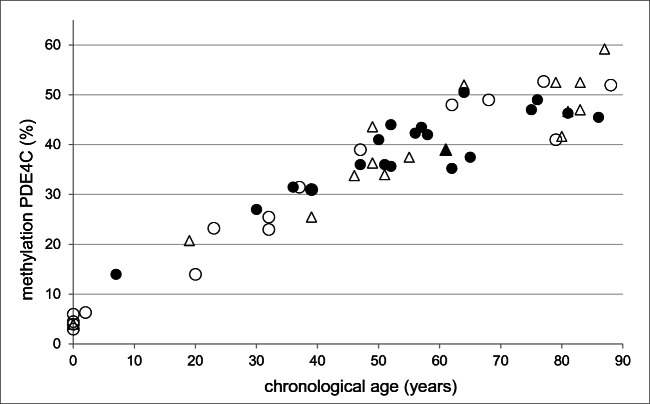
Methylation of PDE4C CpG-1 (%) vs. chronological age in the postmortem samples. Score 1: white circles, *n* = 17, score 2 + 3: white triangles, *n* = 15; score 4 + 5: black circles, *n* = 19; score > 6: black triangles, *n* = 1

To assess the impact of decomposition on methylation-dependent age estimation, the regression of samples within each decomposition state was plotted for the chronological age vs. the degree of *PDE4C* CpG-1 methylation (Fig. 3). There is no indication for a relevant influence of the state of decomposition on the scattering of data. Due to the low number of cases and differences in distribution of chronological ages in each group of decomposition state, further statistical processing was not carried out.

## Discussion

The application of epigenetic age estimation to postmortem cases requires systematic analyses regarding the effects of decomposition. This study focused on the applicability of epigenetic age estimation under different postmortem conditions, including stages of advanced decomposition using buccal swabs as the source of DNA [21].

### DNA yields of swabs were unexpectedly high in the postmortem cases with signs of decomposition (below a score of 6)

In many cases with moderate signs of decomposition, the amount of DNA isolated from corpses was unexpectedly high. This may be because the decomposition processes affect the stability of buccal mucosa. More cells may be available on the swab in deceased individuals with signs of decomposition compared to swabs taken from intact buccal mucosa of living individuals. This hypothesis is supported by the analysis of smear preparations of buccal swabs from corpses in different stages of decomposition (Fig. 1).

Degradation indices over 10 (indicating a possible degradation) were seen in only few cases, surprisingly even in the score groups of advanced decomposition of the bodies. These findings obviously reflect that putrefaction processes differ greatly from one individual to another, depending on many ante- and postmortem factors as bacterial colonization, temperature, and blood congestion or hypostasis.

However, DNA yields of swabs from corpses in very severe stages of decomposition (with scores > 6, see Table 1) were minute in all but one individual. This might not be surprising, as in those stages of decomposition tissues start to dry out. Later on, buccal mucosa is dried out or even nearly totally dissolved.

### Ante- and postmortem samples exhibit a very similar and close correlation between DNA methylation and chronological age and the state of decomposition does not affect the scattering of methylation levels

Eipel et al. [21] addressed the importance of buccal cell composition for the precision of epigenetic age estimation, as it is already known that DNA methylation is tissue- and cell-type dependent. However, their work described that the *PDE4C* marker seems to be rather robust to changes in cell composition (buccal epithelial cells vs. leukocytes). On the other hand, one might have assumed that postmortem *PDE4C* methylation levels may be influenced, changed, or even be unmeasurable due to post- or perimortem cellular processes or bacterial activity. Although only a small number of samples and only one DNA methylation marker was analysed in the present study, the collected data do not give any indication of such influences. Therefore, our data for buccal swabs are in line with results from blood samples of corpses presented in other studies, which also did not find differences in epigenetic age estimations of living vs. deceased [12–15]. By this, we also proved the robustness of the *PDE4C* marker towards changes in cellular composition described by Eipel et al. [21].

## Conclusion

Our data suggest that epigenetic age estimation based on the analysis of buccal swabs is applicable even in cases with advanced decomposition, as long as enough intact DNA can be extracted.

In this “proof of principle” study, we analysed only one type of specimen (buccal swabs), a limited number of samples and only one DNA methylation marker. Hence, systematic postmortem studies including diverse tissues and diverse marker combinations as well as cases with different stages of decomposition are required to exploit the full potential of epigenetic age estimation for postmortem application. Based on the results of such studies, epigenetic age estimation will doubtlessly expand the methodological repertoire for postmortem age estimation. It will be especially valuable in combination with other methods, as such combinations are proven to reduce errors [22].

## Data Availability

All data are available at the Institute of Legal Medicine, University Hospital Düsseldorf, 40225 Düsseldorf, Germany and can be requested from the authors.

## References

[1] Horvath S (2013). DNA methylation age of human tissues and cell types. Genome Biol.

[2] Hannum G, Guinney J, Zhao L, Zhang L, Hughes G, Sadda S, Klotzle B, Bibikova M, Fan JB, Gao Y, Deconde R, Chen M, Rajapakse I, Friend S, Ideker T, Zhang K (2013). Genome-wide methylation profiles reveal quantitative views of human aging rates. Mol Cell.

[3] Levine ME, Lu AT, Quach A, Chen BH, Assimes TL, Bandinelli S, Hou L, Baccarelli AA, Stewart JD, Li Y, Whitsel EA, Wilson JG, Reiner AP, Aviv A, Lohman K, Liu Y, Ferrucci L, Horvath S (2018). An epigenetic biomarker of aging for lifespan and healthspan. Aging (Albany NY).

[4] Weidner CI, Lin Q, Koch CM, Eisele L, Beier F, Ziegler P, Bauerschlag DO, Jockel KH, Erbel R, Muhleisen TW, Zenke M, Brummendorf TH, Wagner W (2014). Aging of blood can be tracked by DNA methylation changes at just three CpG sites. Genome Biol.

[5] Parson W (2018). Age estimation with DNA: from forensic DNA fingerprinting to forensic (Epi)genomics: a mini-review. Gerontology..

[6] Sabeeha, Hasnain SE (2019). Forensic epigenetic analysis: the path ahead. Med Princ Pract.

[7] Naue J, Hoefsloot HCJ, Mook ORF, Rijlaarsdam-Hoekstra L, van der Zwalm MCH, Henneman P, Kloosterman AD, Verschure PJ (2017). Chronological age prediction based on DNA methylation: massive parallel sequencing and random forest regression. Forensic Sci Int Genet.

[8] Jung SE, Lim SM, Hong SR, Lee EH, Shin KJ, Lee HY (2018). DNA methylation of the ELOVL2, FHL2, KLF14, C1orf132/MIR29B2C, and TRIM59 genes for age prediction from blood, saliva, and buccal swab samples. Forensic Sci Int Genet.

[9] Aliferi A, Ballard D, Gallidabino MD, Thurtle H, Barron L, Syndercombe Court D (2018). DNA methylation-based age prediction using massively parallel sequencing data and multiple machine learning models. Forensic Sci Int Genet.

[10] Bajanowski T (2018) Stellungnahme: Forensische Altersdiagnostik bei unbegleiteten minderjährigen Flüchtlingen. https://www.dgrm.de/startseite/news-dgrm/stellungnahme-forensische-altersdiagnostik-bei-unbegleiteten-minderjaehrigen-fluechtlingen/. 20. Mai 2019

[11] Abbott A (2018). European scientists seek 'epigenetic clock' to determine age of refugees. Nature.

[12] Bekaert B, Kamalandua A, Zapico SC, Van de Voorde W, Decorte R (2015). Improved age determination of blood and teeth samples using a selected set of DNA methylation markers. Epigenetics.

[13] Hamano Y, Manabe S, Morimoto C, Fujimoto S, Ozeki M, Tamaki K (2016). Forensic age prediction for dead or living samples by use of methylation-sensitive high resolution melting. Leg Med (Tokyo).

[14] Naue J, Sanger T, Hoefsloot HCJ, Lutz-Bonengel S, Kloosterman AD, Verschure PJ (2018). Proof of concept study of age-dependent DNA methylation markers across different tissues by massive parallel sequencing. Forensic Sci Int Genet.

[15] Dias HC, Cordeiro C, Real FC, Cunha E, Manco L (2019). Age estimation based on DNA methylation using blood samples from deceased individuals. J Forensic Sci.

[16] Lee HY, Hong SR, Lee JE, Hwang IK, Kim NY, Lee JM, Fleckhaus J, Jung SE, Lee YH (2020). Epigenetic age signatures in bones. Forensic Sci Int Genet.

[17] Pfeifer M, Bajanowski T, Helmus J, Poetsch M (2020). Inter-laboratory adaption of age estimation models by DNA methylation analysis-problems and solutions. Int J Legal Med.

[18] Marquez-Ruiz AB, Gonzalez-Herrera L, Luna JD, Valenzuela A (2020). DNA methylation levels and telomere length in human teeth: usefulness for age estimation. Int J Legal Med.

[19] Dias HC, Cordeiro C, Pereira J, Pinto C, Real FC, Cunha E, Manco L (2020). DNA methylation age estimation in blood samples of living and deceased individuals using a multiplex SNaPshot assay. Forensic Sci Int.

[20] Megyesi MS, Nawrocki SP, Haskell NH (2005). Using accumulated degree-days to estimate the postmortem interval from decomposed human remains. J Forensic Sci.

[21] Eipel M, Mayer F, Arent T, Ferreira MR, Birkhofer C, Gerstenmaier U, Costa IG, Ritz-Timme S, Wagner W (2016). Epigenetic age predictions based on buccal swabs are more precise in combination with cell type-specific DNA methylation signatures. Aging (Albany NY).

[22] Becker J, Mahlke NS, Reckert A, Eickhoff SB, Ritz-Timme S (2019). Age estimation based on different molecular clocks in several tissues and a multivariate approach: an explorative study. Int J Legal Med.

